# A lysine-rich cluster in the N-BAR domain of ARF GTPase-activating protein ASAP1 is necessary for binding and bundling actin filaments

**DOI:** 10.1016/j.jbc.2022.101700

**Published:** 2022-02-08

**Authors:** Anjelika Gasilina, Hye-Young Yoon, Xiaoying Jian, Ruibai Luo, Paul A. Randazzo

**Affiliations:** 1 Laboratory of Cellular and Molecular Biology, Center for Cancer Research, National Cancer Institute, National Institutes of Health, Bethesda, Maryland, USA; 2 Department of Biochemistry and Molecular & Cellular Biology, Georgetown University Medical Center, Washington, District of Columbia, USA

**Keywords:** actin, actin-binding protein, microfilaments, cytoskeleton, stress fibers, ADP ribosylation factor, GTPase activating protein, ArfGAP, BAR, Bin/Amphiphysin/Rvs, BSA, bovine serum albumin, CH, calponin homology, F-actin, filamentous actin, PH, pleckstrin homology, SH3, Src homology 3

## Abstract

Actin filament maintenance is critical for both normal cell homeostasis and events associated with malignant transformation. The ADP-ribosylation factor GTPase-activating protein ASAP1 regulates the dynamics of filamentous actin-based structures, including stress fibers, focal adhesions, and circular dorsal ruffles. Here, we have examined the molecular basis for ASAP1 association with actin. Using a combination of structural modeling, mutagenesis, and *in vitro* and cell-based assays, we identify a putative-binding interface between the N-Bin-Amphiphysin-Rvs (BAR) domain of ASAP1 and actin filaments. We found that neutralization of charges and charge reversal at positions 75, 76, and 79 of ASAP1 reduced the binding of ASAP1 BAR-pleckstrin homology tandem to actin filaments and abrogated actin bundle formation *in vitro*. In addition, overexpression of actin-binding defective ASAP1 BAR-pleckstrin homology [K75, K76, K79] mutants prevented cellular actin remodeling in U2OS cells. Exogenous expression of [K75E, K76E, K79E] mutant of full-length ASAP1 did not rescue the reduction of cellular actin fibers consequent to knockdown of endogenous ASAP1. Taken together, our results support the hypothesis that the lysine-rich cluster in the N-BAR domain of ASAP1 is important for regulating actin filament organization.

Regulated and dynamic organization of the actin cytoskeleton plays an important role in guiding cellular processes, such as adhesion, migration, proliferation, differentiation, and survival (1, 2, 3). Organization of monomeric actin and actin filaments into higher order structures is under the control of actin-binding proteins, which fall into many families, such as calponin homology (CH), Wiskott–Aldrich syndrome protein homology domain-2, formin homology 2 (4, 5), and others. Another family of proteins, the Bin-Amphiphysin-Rvs (BAR) domain superfamily, is being actively explored in its regulation of the actin cytoskeleton, although only a handful of its members have been shown to directly bind to actin (6, 7, 8, 9, 10, 11). However, unlike for CH domain protein families, the mechanisms that drive the interactions of the BAR domain proteins with actin are poorly defined.

ASAP1 (also known as DEF1, DDEF1, PAG2, AMAP1, and centaurin β4) is a signaling and adapter protein composed of N-BAR, pleckstrin homology (PH), ArfGAP (Arf GTPase Activating Protein), ankyrin repeats, a stretch of eight E/DLPPKP repeats, a proline-rich, and Src homology 3 (SH3) domains. ASAP1 was first discovered on the basis of GAP activity and association with Src and as a differentiation enhancement factor in adipogenic fibroblasts (12, 13, 14). Loss of ASAP1 *in vivo* leads to partial perinatal lethality and delayed ossification and adipogenesis (15). ASAP1 is implicated in regulating survival, cell motility, and invasiveness in a number of cancers (16, 17, 18, 19). The N-terminal N-BAR domain of ASAP1, a point of dimerization, regulates membrane dynamics and the actin cytoskeleton by interacting with membrane lipids, Rab11 effector FIP3, nonmuscle myosin II A, and actin filaments (20, 21, 22, 23, 24).

We and others independently showed that the BAR-PH region of ASAP1 binds to and bundles actin filaments *in vitro* with similar efficiency as other actin-binding proteins and induces cellular actin remodeling (20, 24). The idea of a specific interaction between the ASAP1 BAR domain and filamentous actin (F-actin) is supported by our observation that exogenous expression of ACAP1, a closely related N-BAR-domain containing ArfGAP, does not rescue the loss of actin structures induced by the reduced expression of ASAP1. However, structural determinants within the BAR domain of ASAP1 that are necessary for F-actin binding have not been defined. Here, guided by homology modeling, we couple cellular and biochemical studies to probe the differences between actin binding to the BAR domain of ASAP1 and binding to the BAR domain of ACAP1 and to define key residue clusters in the BAR domain of ASAP1 that are required for its interaction with the actin filaments. In addition, we examine whether the actin remodeling activity is conserved among the subtypes of the ASAP subgroup of the ArfGAP superfamily.

We reveal that a lysine-rich region located on the BAR domain of ASAP1 is necessary for its actin remodeling activity and that all ASAP subtypes participate in maintaining stress fiber alignment.

## Results

### Actin binding is not conserved between BAR domains of ACAP1 and ASAP1 ARF GAPs

**Figure 1:**
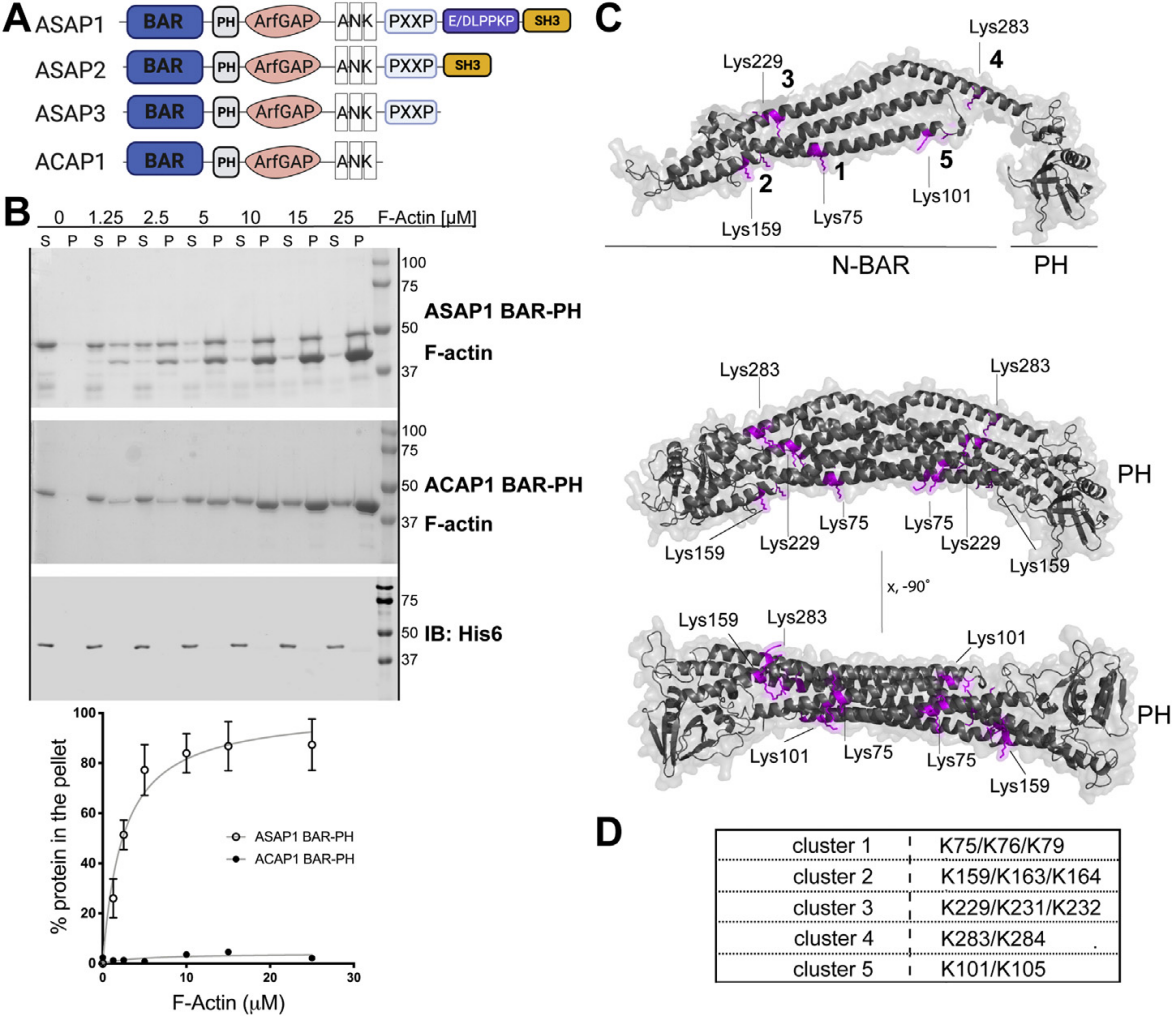
**ACAP1 BAR-PH, unlike ASAP1 BAR-PH, does not bind to the actin filaments.***A*, schematic of the domain architecture of ASAP1, 2, 3, and ACAP1. *B*, results of the high-speed sedimentation assay for purified ASAP1 or ACAP1 BAR-PH in the absence or presence of increasing concentrations of actin filaments. *Top panel* shows increased pelleting of ASAP1 BAR-PH with increasing concentrations of F-actin, and *middle* (GelCode blue stained) and *bottom* (probed with anti-His_6_-antibody to identify ACAP1) panels show no pelleting of ACAP1 BAR-PH. The graph depicts quantification of all experiments. N = 3 independent experiments, mean ± SEM. *C*, surface ribbon representation of the homology model of ASAP1 BAR-PH highlighting lysine-rich clusters (numbered 1–5) in *magenta*. *Top* - monomeric representation of BAR-PH, *middle* and *bottom* - BAR-PH dimer rendering. Structure rendering was performed in PyMOL. *D*, lists residues belonging to each cluster. ANK, ankyrin repeats; BAR, Bin/Amphiphysin/Rvs domain; F-actin, filamentous actin; His_6_, 6x Histidine tag; IB, immunoblot; P, pellet; PH, pleckstrin homology; S, supernatant; SH3, Src homology 3 domain.

To compare the BAR-PH regions of ACAP1 and ASAP1 for binding to F-actin, we performed a high-speed actin sedimentation assay. Purified His_6_-tagged ACAP1(residues 1–370) and ASAP1 (residues 1–431) BAR-PH proteins at 2 μM were incubated with increasing (0–25 μM) concentration of filamentous actin for 30 min at room temperature and subjected to ultracentrifugation at 150,000*g*. Pellets (actin filaments with filament-bound protein) and supernatants (residual globular actin and unbound protein) were resolved on SDS-PAGE. Because the molecular weights of ACAP1 BAR-PH and actin are similar and, therefore, the proteins are difficult to separate by SDS-PAGE, we also detected ACAP1 BAR-PH, fused to hexa-His, by immunoblotting with anti-His_6_ antibody. Pellet fractions were analyzed as a percentage of total test protein added and plotted (% test protein in the pellet). As we previously reported, the amount of ASAP1 BAR-PH in the pellet increased with increasing concentration of actin filaments (20); in contrast, no ACAP1 BAR-PH was found in the pellet with actin filaments (Fig. 1*B*). Both ACAPs and ASAPs belong to the BAR domain superfamily, the members of which share a conserved banana-shaped fold (6); therefore, our observation that *in vitro* actin-binding activity is not shared between ACAP1 and ASAP1 prompted us to investigate if divergence in primary sequence dictates actin remodeling activity of ASAP1.

### Identification of residues involved in interaction with F-actin

Primary literature suggests that some non-CH domain actin-binding proteins, such as pacsin2 (8), palladin (25), PICK1 (26), IRSp53 (27), and fascin (28) employ basic patches, at least in part, in their interaction with the actin filaments.

Our original efforts to obtain high-resolution crystal structure of ASAP BAR-PH failed due to inability to obtain diffraction-quality crystals despite our many attempts.† In the absence of experimentally derived structural information, we built a homology model of ASAP1 BAR-PH using crystal structure of ACAP1 BAR-PH as a template (24).

Five lysine-rich clusters were identified from the homology model, indicated as 1 to 5 (Fig. 1, *C* and *D*). To test for a role in actin binding, we introduced mutations into cDNA that would result in replacing lysine with glutamate (charge reversal) or alanine (charge neutralization) (Fig. S1*B*). Of the mutants generated, six of nine were useful for testing the hypothesis. Two mutants, [K101A, K105A]ASAP1-BAR-PH-His_6_ and [K159A, K163A, K164A] ASAP1-BAR-PH-His_6_, did not express despite varying induction temperature and duration, media formulation, and IPTG concentration. For the rest of the mutants, we assessed changes in stability by conducting gel-based thermal stability assay, which allows for robust quantitative assessment of protein behavior (29). The melting temperature for [K229E, K231E, K232E] ASAP1-BAR-PH-His_6_ triple mutant could not be determined, and this mutant was not used for further analysis. The melting temperatures of [K75E, K76E, K79E] ASAP1-BAR-PH-His_6_, [K75A, K76A, K79A] ASAP1-BAR-PH-His_6_ triple mutants, [K283E, K284E] ASAP1-BAR-PH-His_6_ double mutant, [K283A, K284A] ASAP1-BAR-PH-His_6_ double, and [K229A, K231A, K232A] ASAP1-BAR-PH-His_6_ triple mutant were similar to WT protein ranging from 49 to 52 °C, and the melting temperature of [K159E, K162E, K164E] ASAP1-BAR-PH-His_6_ was 47 °C ± 0.5 deg. C, somewhat lower than other mutants or the WT protein. These six mutants were used for further analysis.

**Figure 2:**
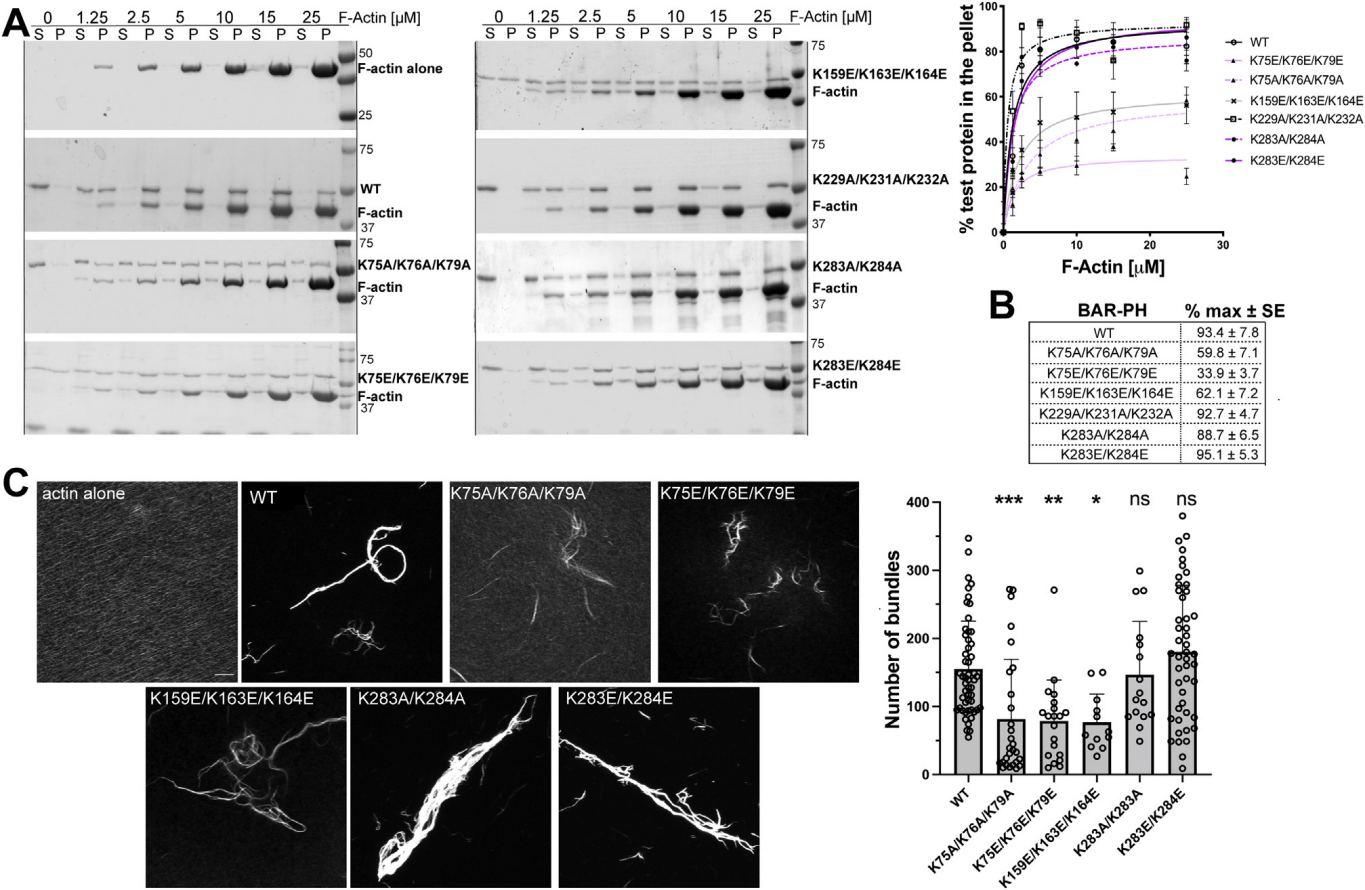
**Basic patch involving amino acids 75, 76, and 79 on ASAP1 BAR-PH is critical for binding and bundling F-actin.***A* and *B*, results of high-speed actin cosedimentation experiments show control actin filament sedimentation (F-actin alone), cosedimentation of actin filaments with WT ASAP1 BAR-PH (WT), [K229A, K231A, K232A], [K283A, K284A], and [K283E, K284E] mutants. Cosedimentation of ASAP1 [K75A, K76A, K79A], [K75E, K76E, K79E], and [K159E, K163E, K164E] with actin filaments is impaired. Quantification of actin filament binding by ASAP1 BAR-PH mutants and maximal binding are presented in the graph (*A*, % test protein in the pellet, mean ± SEM) and in tabular form (*B*, % max and SE), respectively. N = 2 to 3 independent experiments. *C*, representative images of fluorescence-based actin bundling assay in the absence or presence of ASAP1 BAR-PH, and mutants and graph summarizing quantification of the number of bundles (mean ± SD) induced by each protein. F-actin was incubated alone or with BAR-PH on glass coverslips and the bundles were stained with fluorescent phalloidin and assessed using confocal microscopy. The scale bar represents 10 μm, N = 3 independent experiments with 10 to 15 replicates. BAR, Bin/Amphiphysin/Rvs domain; F-actin, filamentous actin; ns, not significant; P, pellet; PH, pleckstrin homology; S, supernatant. ∗*p* < 0.05; ∗∗*p* < 0.01; ∗∗∗*p* < 0.001.

To evaluate the effects of mutations on binding to the actin filaments, we performed high-speed actin cosedimentation assays (Fig. 2, *A* and *B*). Cluster 1 mutants and cluster 2 mutants bound less efficiently than did the WT protein, with the mutant [K75E, K76E, K79E] having the most severe defect. Other mutants bound F-actin to the same extent as WT. These results suggested that the lysine clusters [75, 76, 79] and [159, 163, 164] were important for binding to the actin filaments.

We then assessed the effects of the mutations on the ability of ASAP1 BAR-PH to crosslink actin filaments into bundles. Using a fluorescence-based actin bundling assay, wherein formed bundles can be visualized by fluorescent phalloidin staining, we determined that both Ala and Glu triple mutants of cluster 1 [K75, K76, K79] produced fewer bundles than BAR-PH WT (Fig. 2*C*). Cluster 2 [K159E, K163E, K164E] ASAP1-BAR-PH-His_6_ triple mutant also had reduced bundling efficiency. As an independent control, we used the [K283, K284] double mutants. We hypothesized that since this cluster is not important for actin binding based on our high-speed sedimentation data, mutating these residues should not affect the formation of actin bundles. Indeed, both [K283A, K284A] ASAP1-BAR-PH-His_6_ and [K283E, K284E] ASAP1-BAR-PH-His_6_ double mutants formed bundles that were comparable to the WT protein.

### Lysine clusters 1 (K75, K76, K79) and 2 (K159, K163, K164) are necessary for effects of ASAP1-BAR-PH in cells

**Figure 3:**
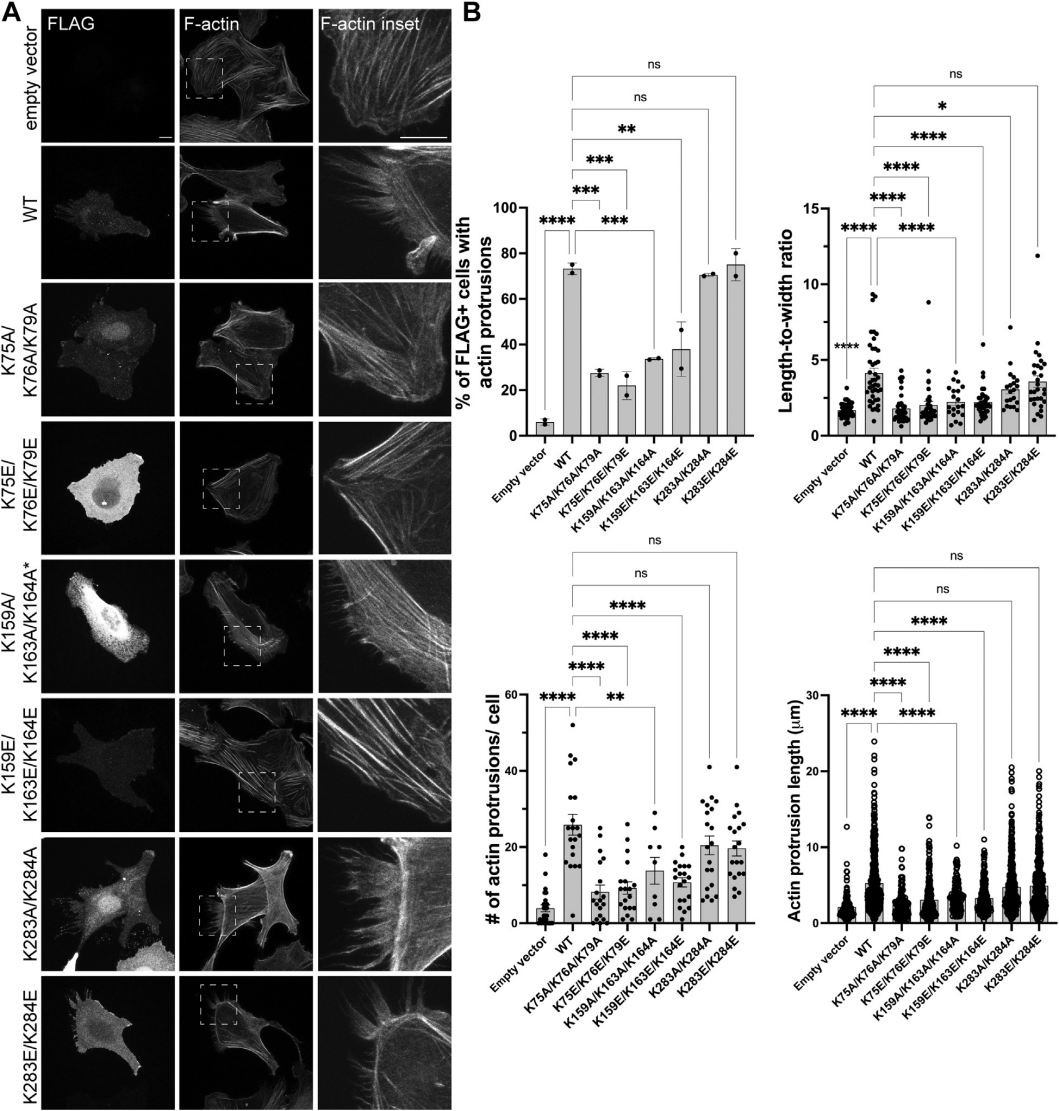
**The effect of mutations on cellular actin reorganizing activity induced by BAR-PH.** Overexpression of FLAG ASAP1 BAR-PH WT, [K283A, K284A], and [K283E, K284E], but not [K75A, K76A, K79A], [K75E, K76E, K79E], [K159A, K163A, K164A], and [K159E, K163E, K164E], causes formation of actin protrusions. Human osteosarcoma cells U2OS were transiently transfected with empty vector, or FLAG-tagged 1 to 431 (BAR-PH) WT and indicated mutants and costained with anti-FLAG antibody and fluorescent phalloidin for F-actin. Images are representative of two independent experiments with at least 20 cells per group per experiment, except ∗[K159A, K163A, K164A] mutant, which showed low expression (5–6 cells). Insets highlight the presence or absence of actin protrusions. The scale bars (main and inset) represent 10 μm. *B*, graphs summarizing the quantification of percentage of BAR-PH expressing cells that contain long actin protrusions, quantification of shape changes (length-to-width ratio) induced by BAR-PH expression, number of actin protrusions per cell, and average length of the protrusions per cell, presented as mean ± SD. Random empty vector cells were counted as a reference. Statistics are based on the quantification of cells from all experiments using One-way ANOVA with Dunnet’s multiple comparison test (compared to the WT BAR-PH), n.s., not significant, ∗*p* < 0.05; ∗∗*p* < 0.01; ∗∗∗*p* < 0.001; ∗∗∗∗*p* < 0.0001. BAR, Bin/Amphiphysin/Rvs domain; F-actin, filamentous actin; PH, pleckstrin homology.

We then examined the effect of the mutations on cellular behavior of ASAP1 BAR-PH. Previously, we and others independently showed that exogenous expression of the isolated BAR-PH region of ASAP1 leads to deregulated bundling of actin, resulting in numerous actin-rich protrusions and microspikes (24), thickening of cortical actin, and elongated cell shape (20). We introduced mutations into the cDNA in a mammalian expression vector for FLAG epitope-tagged ASAP1 BAR-PH that changes lysines in clusters 1, 2, and 4 as indicated in Figure 3. The proteins were expressed in a human osteosarcoma cell line U2OS, which is characterized by its well-defined actin stress fiber morphology, to compare the effect of mutants to the WT BAR-PH. We transfected the cells with the empty vector or FLAG-tagged WT BAR-PH or mutants, replated the cells on fibronectin 24 h posttransfection, and costained them with anti-FLAG antibody and fluorescent phalloidin for filamentous actin. As shown in Figure 3*A*, F-actin and F-actin inset panels, exogenous expression of WT BAR-PH (WT) produced characteristic F-actin-filled protrusions, with 73 ± 2% of FLAG-positive cells displaying actin protrusions (Fig. 3*B*) compared to 6 ± 1% of cells transfected with the empty vector. In contrast, cells expressing the FLAG-ASAP1 [K75A, K76A, K79A, 1–431] and FLAG-ASAP1 [K75E, K76E, K79E, 1–431] triple mutants led to distinct actin protrusions in only 27 ± 1% and 22 ± 4% of cells, respectively. The number and length of actin protrusions per cell was also reduced (Fig. 3*B*).

Cells expressing FLAG-[K159A, K163A, K164A] ASAP1-BAR-PH and FLAG-[K159E, K163E, K164E] ASAP1-BAR-PH had actin protrusions in 34 ± 0.3% and 38 ± 9% of the cells, respectively. We also noted that despite many transfection attempts using several plasmid preparations, the efficiency of expressing FLAG-[K159A, K163A, K164A] ASAP1-BAR-PH was less than 15% than that of the other mutants. Combined with the lack of soluble recombinant expression for this mutant, this result indicates that [K159, K163, K164] residue cluster may be important for folding and stability of ASAP1 BAR-PH or its expression may also be toxic to cells.

The double mutants [K283A, K284A]ASAP1-BAR-PH and [K283E, K284E]ASAP1-BAR-PH, which did not display any defect in *in vitro* assays of actin binding and bundling also did not display any appreciable defect on cellular actin remodeling. Representative images for both charge neutralizing A and charge reversal E mutants show robust actin-filled protrusions (Fig. 3*A*) in U2OS cells, with 71 ± 1% and 75 ± 5% of FLAG-positive cells exhibiting actin protrusions (Fig. 3*B*). The cells overexpressing mutants presented with varying degrees of cell shape changes, as judged by the length-to-width ratios. Wild type and [K283, K284]ASAP1-BAR-PH mutants had similar increases in length (cell elongation) compared to the empty vector expressing cells, while [K75, K76, K79]ASAP1-BAR-PH and [K159, K163, K164]ASAP1-BAR-PH triple mutant-expressing cells exhibited cell shape comparable to those transfected with the empty vector plasmid.

Taken together, our *in vitro* biochemical and cellular data suggest that cluster 1 [K75, K76, K79] and cluster 2 [K159, K163, K164] are important for the interaction of ASAP1 BAR-PH with filamentous actin.

### Residues adjacent to the primary [K75, K76, K79] cluster participate in actin filament interactions

**Figure 4:**
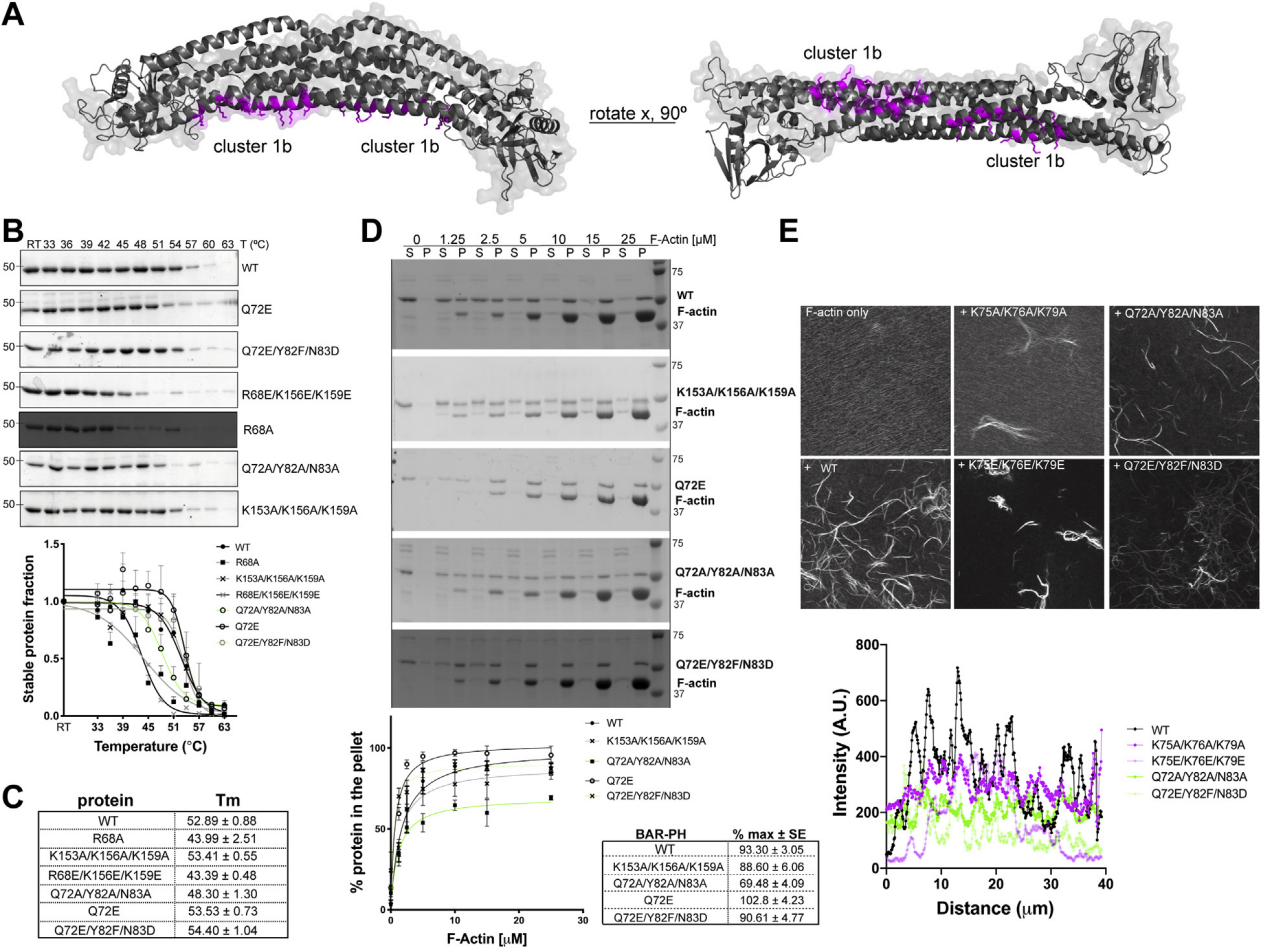
**Amino acids surrounding primary basic patch [K75, K76, K79] contribute to actin bundling activity of BAR-PH.***A*, surface ribbon representation of the homology model of ASAP1 BAR-PH highlighting lysine-rich cluster 1b in *magenta*. *B*, thermal stability assay of subsequent set of mutants was performed as described for Figure 2 and Fig. S1. *C* and *D*, high-speed cosedimentation assays were performed for mutants whose stability was not affected by mutations. Results of high-speed actin cosedimentation experiments show control actin filament sedimentation (F-actin alone) and cosedimentation of actin filaments with WT ASAP1-His_6_ BAR-PH, [Q72E, Q72E, Y82F] ASAP1-His_6_. Cosedimentation of ASAP1 BAR-PH [Q72A, Y82F, N83A] ASAP1-His_6_ is mildly impaired. Experiments were performed as in Figure 2*A*. Mean ± SEM. Quantification of actin filament binding by ASAP1 BAR-PH mutants and maximal binding are presented in the table (*C*, % max and SE) and in the graph (*D*, % protein in the pellet, mean ± SEM). N = 3 independent experiments. *E*, images from the fluorescence-based actin-bundling assay in the absence or presence of ASAP1 BAR-PH and mutants. F-actin was incubated alone or with BAR-PH on glass coverslips and the bundles were stained with fluorescent phalloidin and assessed using confocal microscopy. Direct comparison of initially identified and further refined basic patch show reduced actin bundling by Q72/Y82/N843 mutants. The graph summarizes line scan intensities of actin bundles formed by WT BAR-PH or the indicated mutants. The scale bar represents 10 μm, N = 2 independent experiments, two technical replicates each, using LAS X Navigator tile scanning, 50 tiles per replicate. BAR, Bin/Amphiphysin/Rvs domain; F-actin, filamentous actin; P, pellet; PH, pleckstrin homology; S, supernatant.

Having identified a role for cluster 1 for the effect of ASAP1 BAR-PH on actin *in vitro* and cells, we sampled around this patch to probe if adjacent residues participate in the interaction with the actin filaments. The second round of mutants, single mutants Q72E, R68A and triple mutants [Q72E, Y82F, N83D] ASAP1-BAR-PH-His_6_, [Q72A, Y82A, N83A] ASAP1-BAR-PH-His_6_, [R68E, K156E, K159E] ASAP1-BAR-PH-His_6_, and [K153A, K156A, K159A] ASAP1-BAR-PH-His_6_, designated as cluster 1b in Figure 4*A* were subjected to thermal stability assays as before. As shown in Figure 4*B*, R68A single mutant had a significantly lower melting temperature (T_m_) than the WT BAR-PH in this assay: 44 °C ± 3 deg. C *versus* 53 °C ± 1 deg. C. [R68E, K156E, K159E] ASAP1-BAR-PH-His_6_ triple mutant likewise exhibited decreased stability, with a melting temperature of 43 °C ± 0.5 deg. C. Therefore, these two mutants were not considered for further characterization. The melting temperatures of [K153A, K156A, K159A] ASAP1-BAR-PH-His_6_, [Q72A, Y82A, N83A] ASAP1-BAR-PH-His_6_, [Q72E, Y82F, N83D] ASAP1-BAR-PH-His_6_ triple mutants, and Q72E single mutant were comparable to that of the WT BAR-PH (Fig. 4*C*).

The mutants in the resulting group were assessed for their ability to bind and bundle filamentous actin (Fig. 4*D*). We found that maximum binding of [Q72A, Y82A, N83A] ASAP1-BAR-PH-His_6_ was 30% less than that of WT protein. Other mutants were similar to WT protein. We then used a fluorescence-based actin bundling assay to compare cluster 1a [K75, K76, K79] and cluster 1b [Q72, Y83, N83] side-by-side. As shown in Figure 4*E*, incubation of WT BAR-PH with actin filaments leads to cross-linking of filaments into bundles. In the presence of charge reversing or charge neutralizing [K75, K76, K79] triple mutant, actin filaments do not appear to bundle efficiently, as we also present in Figure 2*B*, appearing as short loose patches. In the presence of [Q72A, Y82A, N83A] ASAP1-BAR-PH-His_6_ triple mutant, which had mild decrease in its ability to bind actin filaments, cross-linking of actin was also inefficient, with fewer bundles per field than the WT BAR-PH. [Q72E, Y82F, N83D] ASAP1-BAR-PH-His_6_ triple mutant, which exhibited no defects in actin binding activity, produced long, but qualitatively less bright bundles, indicating a defect in cross-linking activity. Using the confocal laser scanning microscopy images from the bundling assay, we determined fluorescent intensity across bundles as a readout of bundling efficiency. We plotted the fluorescence intensity of line scans across individual bundles to quantify differences in actin bundling activity of mutants. As summarized in intensity line scan plot in Figure 4*E*, compared to the WT BAR-PH (black line), mutants of both [K75, K76, K79] and [Q72, Y83, N83] clusters produced actin bundles of lower fluorescence intensity (magenta and green lines).

Taken together these data indicate that [Q72, Y83, N83] cluster, adjacent to the [K75, K76, K79] cluster, aids in cross-linking of actin filaments into bundles.

### Full-length ASAP1 with [K75E, K76E, K79E] mutation is unable to rescue the reduction in actin stress fibers induced by the loss of endogenous ASAP1

**Figure 5:**
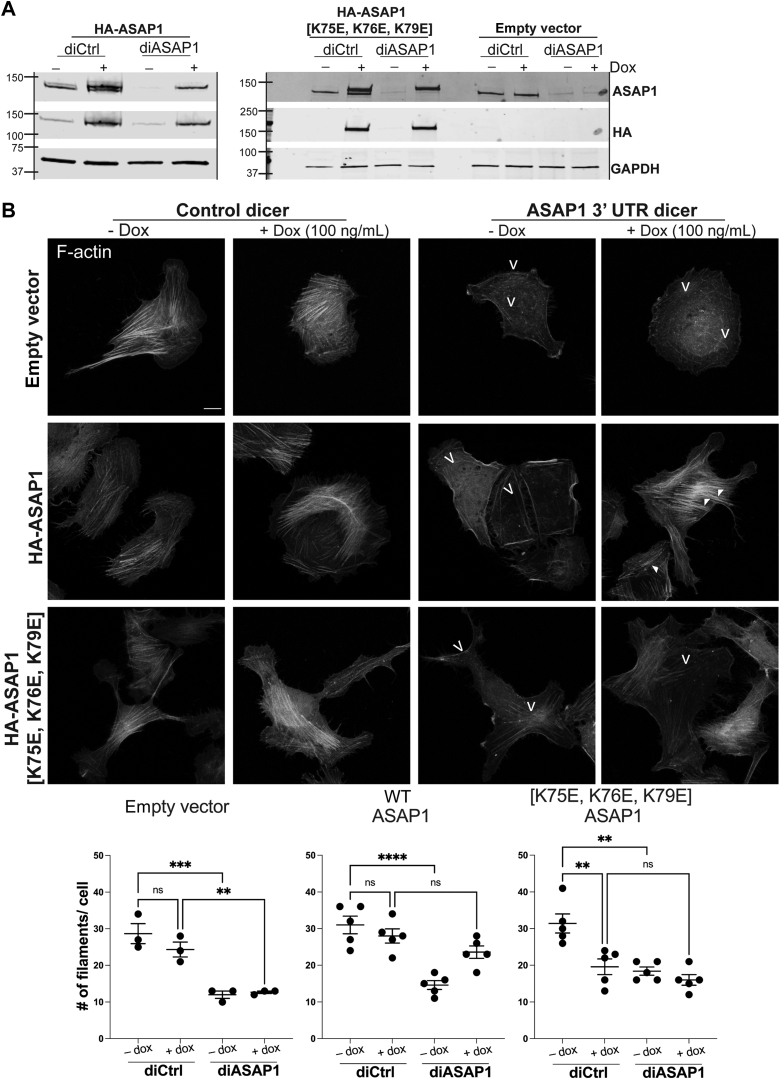
**Complementation with ASAP1 [K75E, K76E, K79E] does not rescue the effects of ASAP1 downregulation on actin stress fibers.** U2OS cells were stably transduced with tet-inducible empty vector, full-length WT (HA-ASAP1), or HA-ASAP1 [K75E, K76E, K79E] lentiviruses and transfected with control (diCtrl) DICER substrate RNA duplex or diRNA against the 3′UTR region of human ASAP1 (diASAP1). After 24 h, expression of the empty vector or ASAP1 was induced or not with doxycycline (100 ng/ml) for 48 h. The cells were subsequently processed for immunoblotting or immunofluorescence experiments. *A*, immunoblot summarizing the efficiency of knockdown of endogenous ASAP1 and doxycycline-induced overexpression of empty vector or ASAP1 and mutants. Membranes were probed with rabbit anti-ASAP1 antibody to detect endogenous and exogenous ASAP1, mouse anti-HA antibody to detect expression of transgenes, and anti-mouse GAPDH as a loading control. *B*, cells were plated on fibronectin in serum-free media, fixed and stained with fluorescent phalloidin for F-actin. After 5.5 h in serum-free media, U2OS develop a robust stress fiber network (diCtrl), which is greatly diminished in diASAP1-treated cells. *Open arrowheads* indicate the loss or reduction of stress fibers, whereas *closed arrowheads* indicate rescue of stress fibers. The graphs summarize the quantification of the number of actin filaments per cell in each treatment group from individual experiments. The scale bar represents 10 μm. N = 3 independent experiments for empty vector control and five independent experiments for WT and mutant ASAP1, with 10 to 30 cells for each condition. See also Fig. S1. Statistics are based on One-way ANOVA with Dunnet’s multiple comparison test n.s. – not significant, ∗∗*p* < 0.01, ∗∗∗*p* < 0.001, ∗∗∗∗*p* < 0.0001. Dox, doxycycline; F-actin, filamentous actin.

Our results with truncated proteins both *in vitro* and in cells indicated that the lysine rich cluster including K75, K76, and K79 was critical for ASAP1 regulation of actin. To further test the hypothesis, we examined the activity of ASAP1 and the mutant to support stress fiber formation in cells (20, 22). Using lentivirus-mediated transduction, we created stable U2OS cell lines, expressing empty vector, HA epitope tagged full-length (open reading frame only) WT ASAP1 (HA-ASAP1), or HA-ASAP1 [K75E, K76E, K79E] under a tetracycline-inducible promoter. We transfected the cell lines with control DICER substrate RNA duplex (diCtrl) or RNA duplex against the 3′UTR of ASAP1 (diASAP1). After 24 h, we induced the expression of empty vector and HA-tagged ASAP1 proteins with doxycycline or left the cells uninduced. After an additional 48 h, the cells were harvested for immunoblotting or processed for immunofluorescence. To assess knockdown efficiency, the cell lysates were resolved on SDS-PAGE, transferred on nitrocellulose membranes and probed with ASAP1 antibody to check knockdown efficiency, anti-HA tag antibody to check the levels of transgene induction in the presence of doxycycline, and GAPDH as a loading control. Immunoblot in Figure 5*A* shows efficient knockdown of ASAP1 using ASAP1 diRNA (diASAP1) across all cell groups (panel ASAP1). Addition of doxycycline (+) induces expression of the WT or mutant ASAP1 proteins (panel HA). Appearance of doublet bands in control diRNA-treated cells (diCtrl) in panels probed with ASAP1 antibody indicates endogenous ASAP1 and transgenic ASAP1 expression.‡

To assess the effect of transgenes on stress fiber organization, the cells were replated on fibronectin-coated coverslips for 5.5 h in serum-free media, fixed, stained with fluorescent phalloidin, and imaged by confocal microscopy. We then quantified the number of actin fibers greater than 2.2 μm in length per cell using the Ridge Detection plugin in ImageJ (see “Experimental procedures”) (30). Cells stably transduced with empty vector and transfected with control diCtrl show robust stress fiber network with or without addition of doxycycline (Fig. 5*B*, panel empty vector). Knockdown of ASAP1 (diASAP1) leads to reduction of stress fibers (open arrowheads), and this effect cannot be rescued by inducing the expression of the empty vector with doxycycline (Figs. 5*B* and S2). In comparison, expression of WT full length ASAP1 (HA-ASAP1) in cells treated with ASAP1 diRNA and doxycycline rescues the stress fiber network (Fig. 5*B*, panel HA-ASAP1, closed arrowheads). Expression of cluster 1 mutant full-length ASAP1 (HA-ASAP1 [K75E, K76E, K79E]) did not rescue the loss of stress fibers; instead, the mutant may have functioned as a dominant negative reducing actin fiber formation in cells having endogenous ASAP1 present. Taken together, these results suggest that the BAR domain of ASAP1 is required for the maintenance and development of stress fibers and further support our hypothesis that [K75, K76, K79] cluster is important for ASAP1 interaction with actin.

### *In vitro* actin-binding and -bundling activity is conserved among BAR-PH regions of ASAP ARF GAPs

ASAP proteins fall into the ArfGAP family of proteins. Three main subtypes — ASAP1 (DEF1, DDEF1, centaurin β4, PAG2, or AMAP1), ASAP2 (PAP, PAG3, or DDEF2), and ASAP3 (DDEFL1 or UPLC1) — are known to exist. All three contain N-BAR, PH, ArfGAP, ankyrin repeat, and proline rich domain, but ASAP1 and 2 also contain C-terminal SH3 domain. Both ASAP1 and ASAP3 associate with focal adhesion and circular dorsal ruffles and control levels of filamentous actin in cells, but very little is known about the role of ASAP2 in the regulation of the actin cytoskeleton (31, 32, 33, 34).

We posited that if ASAP subtypes have high level of amino acid conservation in their BAR domains, they would similarly bind actin filaments. We performed ClustalW alignment of the BAR-PH regions of human and mouse ASAPs. While ASAP2-BAR-PH and ASAP3-BAR-PH were 52 and 58% identical to ASAP1, the residues determined important for binding to the actin filaments were 100% identical or highly conserved among ASAP1, ASAP2, and ASAP3 (Fig. S3, pink shading).

**Figure 6:**
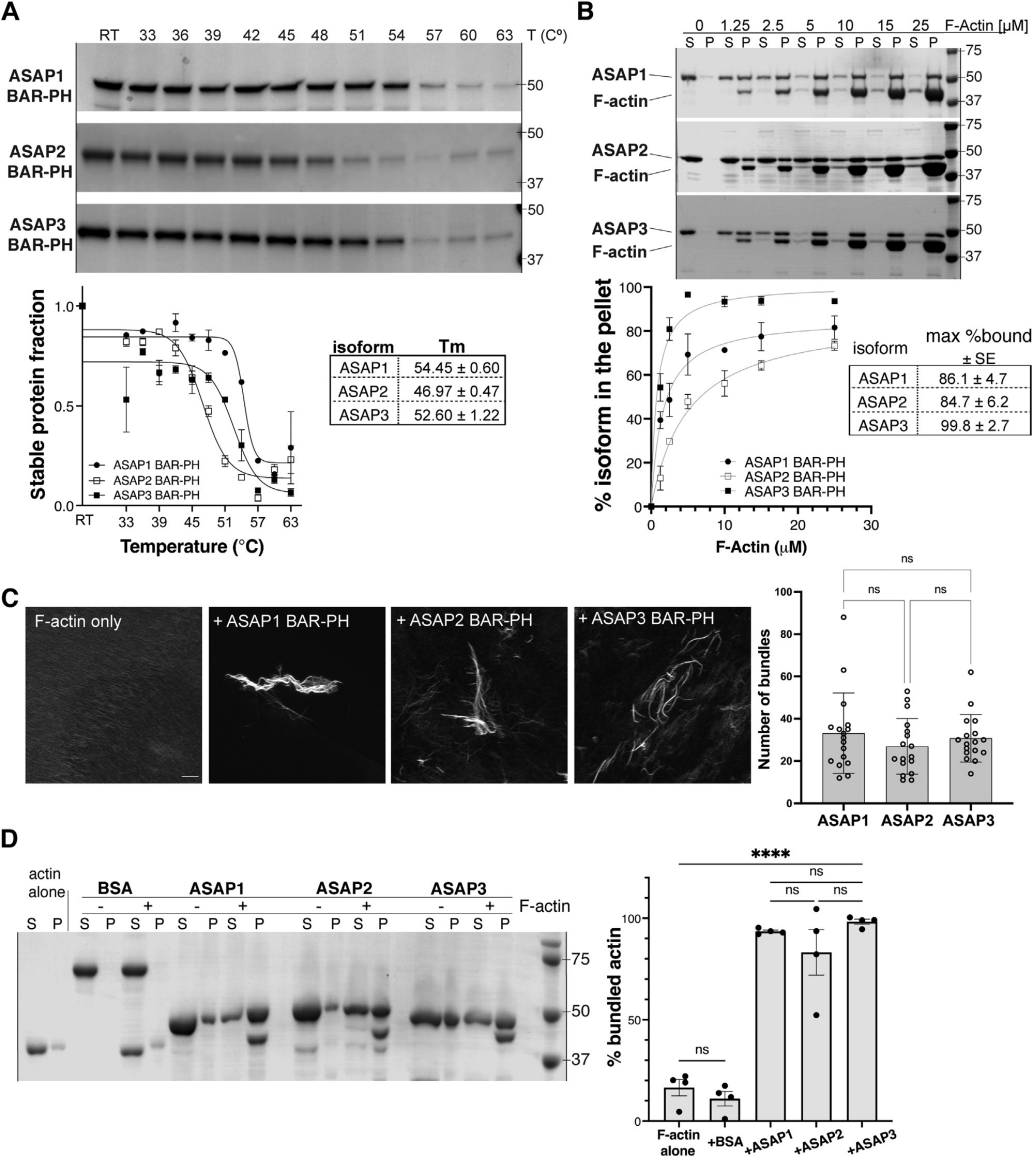
***In vitro* actin filament binding and bundling activity of BAR-PH region is conserved in ASAP subtypes.***A*, thermal stability of purified BAR-PH of ASAP1 — three was assessed as in Fig. S1. *B*, results of high-speed cosedimentation assay show the sedimentation of actin filaments with BAR-PH of ASAP1, ASAP2, and ASAP3, with a graph summarizing the quantification of actin binding (% protein in the pellet, mean ± SEM) and a table presenting approximate binding maxima. N = 2. *C*, results of fluorescence-based actin bundling assay in the absence or presence of ASAP1, ASAP2, or ASAP3. The images are representative of two independent experiments. The scale bar represents 10 μm. The graph summarizes quantification of the number of bundles produced by the BAR-PH regions of ASAP1, 2, or 3 (mean ± SD). *D*, results of actin low-speed cosedimentation-based bundling assays in the absence or presence of BSA (negative control), ASAP1, ASAP2, or ASAP3 BAR-PH. F-actin was sedimented alone (actin alone) at low speed as a control for aggregation. Bovine serum albumin and ASAPs were sedimented alone or in the presence of actin filaments, and bundle formation (% bundled actin) was quantified using densitometric analysis. The graph summarizes the results from four independent experiments, mean ± SD. n.s. – not significant, ∗∗∗∗*p* < 0.0001 using One-way ANOVA with Dunnet’s multiple comparison test. BAR, Bin/Amphiphysin/Rvs domain; BSA, bovine serum albumin; F-actin, filamentous actin; PH, pleckstrin homology.

Prior to high-speed sedimentation assays with the actin filaments, we assessed the thermal stability of the purified BAR-PH regions of ASAP1, ASAP2, and ASAP3. ASAP1-BAR-PH and ASAP3-BAR-PH exhibited melting temperature of 54.5 °C ± 0.6 deg. C and 52.6 °C ± 1.2 deg. C, respectively, while ASAP2-BAR-PH exhibited a much lower melting temperature than the other subtypes, of 47.0 °C ± 0.5 deg. C (Fig. 6*A*). We determined binding to F-actin as in Figures 1, 2, and 4. Half-maximal binding of ASAP1 and ASAP3 occurred at ∼1 μM actin, while half maximum binding of ASAP2 was seen with ∼4 μM. There were modest differences in binding maxima with 100 ± 3% of ASAP3, 86 ± 5% of ASAP1, and 85 ± 6% of ASAP2 sedimenting with actin. We noted that unlike ASAP1 and ASAP2, both mouse and human ASAP3 BAR-PH have an arginine and not a lysine at positions 79, 156, and 159, although the effects could also be explained by heterogeneous populations of proteins in the prep or varying stability.

To test for the conservation of actin bundling activity, we used two independent assays: a fluorescence-based and a low-speed cosedimentation assay, with the latter providing a more quantitative means of assessing differences in actin cross-linking. The three subtypes efficiently cross-link actin filaments into bundles as observed by fluorescent microscopy (Fig. 6*C*). Similarly, the three subtypes did not have detectable differences in their ability to cross-link actin filaments as assessed by the low-speed cosedimentation assay (Fig. 6*D*). Taken together, these data indicate that *in vitro* actin binding and bundling activity is conserved in the BAR domains of ASAP subtype Arf GAPs.

### All three ASAP subtypes contribute to stress fiber alignment in U2OS osteosarcoma cells

**Figure 7:**
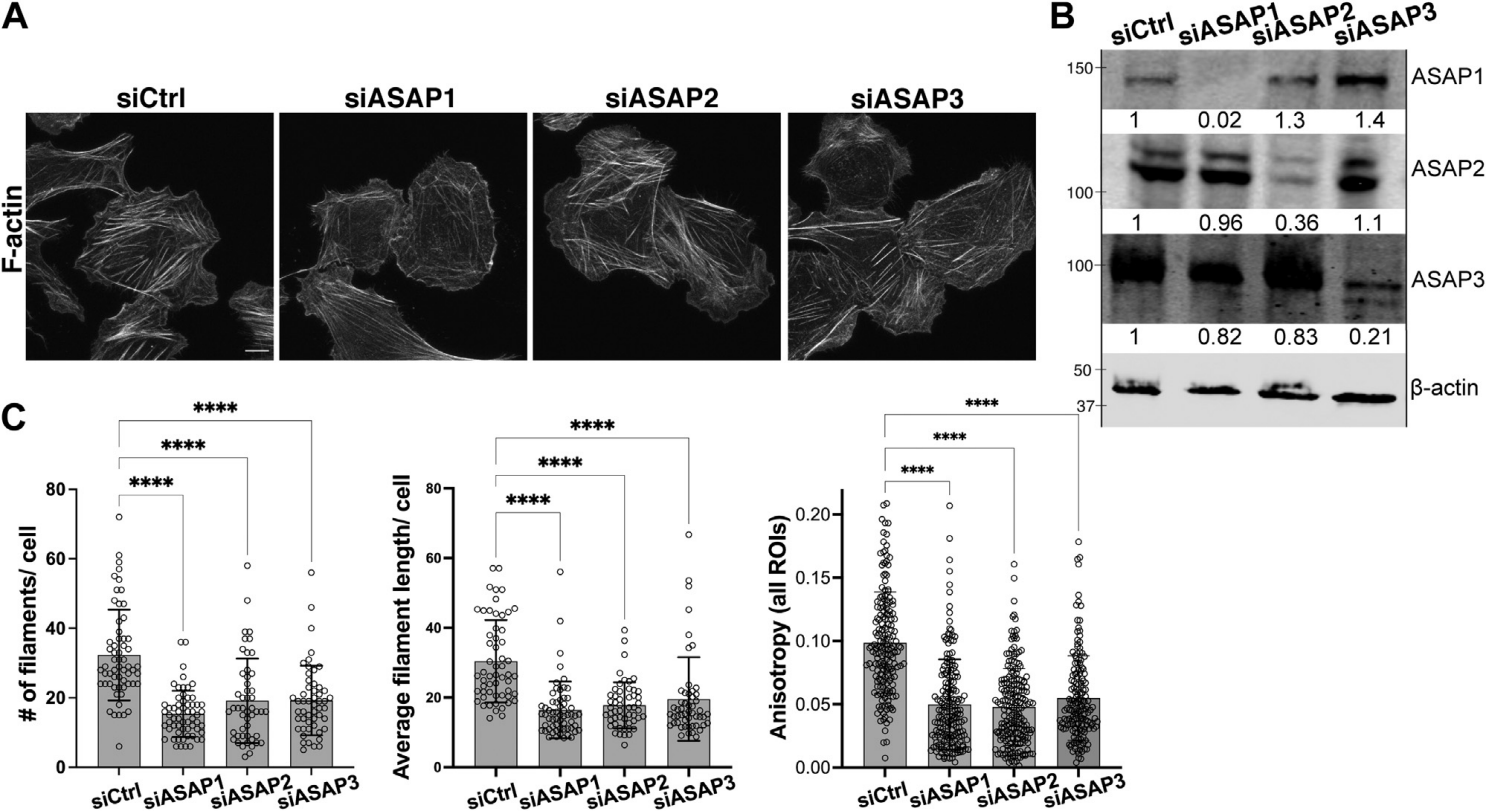
**Downregulation of ASAP subtypes leads to misalignment of stress fibers in U2OS cells.** Human osteosarcoma U2OS cells were transiently transfected with control or pool of siRNAs against ASAP1, ASAP2, or ASAP3. After 72 h, the cells were plated on fibronectin-coated coverslips, stained with fluorescent phalloidin for F-actin, and analyzed by confocal microscopy. *A*, fluorescent images show partial loss of stress fibers induced by knockdown of ASAPs. The scale bar represents 10 μm. *B*, immunoblot and quantification confirm the knockdown of ASAP1, 2, or 3 by their respective siRNA pools. Efficiency of knockdown and off-target effects induced by siRNAs (values below each lane) were quantified relative to β-actin loading control. *C*, the graphs (mean ± SD) summarize the quantification of actin filaments per cell, average filament length, and fiber misalignment induced by downregulation of ASAP expression. Alignment of stress fibers (anisotropy) was quantified using FibrilTool plugin as described in Experimental procedures and plotted as combined results of all experiments. N = 3 independent experiments with 15 to 20 cells each. ∗∗∗∗*p* < 0.0001 using One-way ANOVA with Dunnet’s multiple comparison test. F-actin, filamentous actin; ROI, region of interest.

We have previously shown that depletion of ASAP1 leads to loss and misalignment of stress fibers in mouse and human fibroblasts and U2OS cells (20, 22), and ASAP3 loss leads to the disruption of stress fibers in MDA-MB-231 and U118 cells (33). Here, we directly compared the effect of all three ASAP subtypes on stress fiber assembly. We individually depleted ASAP1, ASAP2, or ASAP3 in U2OS human osteosarcoma cells using SMARTpool siRNAs, replated the cells on fibronectin in serum-free media, stained the cells with fluorescent phalloidin, and examined them using laser scanning confocal microscopy. Compared to cells transfected with control nontargeting siRNA (siCtrl), cells, in which ASAP1, ASAP2, or ASAP3 levels were depleted, had fewer and shorter stress fibers (Fig. 7*C*). We then evaluated stress fiber alignment using FibrilTool, which quantifies organization and anisotropy of fibrillar structures, such as actin stress fibers, where a score of 0 indicates no order or alignment and score of 1 indicates perfectly parallel fibrillar structures (35). The cells transfected with control siRNA had anisotropy score of 0.10 ± 0.003. In contrast, cells transfected with ASAP1 siRNA had anisotropy score of 0.05 ± 0.003, siASAP2 cells had anisotropy score of 0.05 ± 0.002, and cells transfected with ASAP3 siRNA had anisotropy score of 0.06 ± 0.003 (Fig. 7*C*). Thus, ASAP1, ASAP2, and ASAP3 play a role in the maintenance of actin stress fiber organization.

## Discussion

In this work, we continue to test the hypothesis that ASAP1 specifically binds F-actin to regulate stability and alignment of stress fibers. We mapped the putative binding interface between F-actin and the BAR domain of ASAP1 using homology and mutagenesis. We found a cluster of positively charged residues conserved in ASAP subtype Arf GAPs, but not in the highly related ACAP family, that is necessary for actin binding *in vitro* and in cells and for ASAP1 function as a regulator of stress fiber maintenance. ASAP1 functions in processes ranging from cell migration, cancer cell invasion, and immune inflammation (15, 16, 17, 18, 22, 31, 36, 37, 38, 39). Regulation of the actin cytoskeleton has been hypothesized to be important to these functions. Identification of these mutants will thus provide important tools for testing the related hypotheses.

The primary goal of this work was to identify structural determinants within the BAR-PH domain tandem of ASAP1 that are critical for binding and bundling actin filaments. In our previous work, which focused on identifying which domain of ASAP1 is responsible for interaction with actin filaments, we unexpectedly observed that a related BAR domain-containing ArfGAP ACAP1 did not exhibit the same effects on cellular actin and was not able to rescue the defects on actin organization induced by ASAP1 loss. Here, we further confirm that ACAP1 BAR-PH, unlike that of ASAP1, does not bind to actin filaments *in vitro*. The differences between ASAPs and ACAPs in controlling actin organization further highlight already noted diversity in functions among the members of the BAR domain superfamily (6).

Although best described as the sites for membrane binding and bending (40, 41, 42, 43), one of the first BAR domain family members was characterized as a protein-interacting module (44, 45, 46), and several BAR domain-containing proteins have since been characterized as actin-binding proteins (7, 8, 9, 27). This prompted us to investigate the unique features of ASAP1 and ASAP subtype at large that afford them the actin reorganizing activity through their BAR domain.

We used homology modeling and mutagenesis to identify potential structural determinants of actin binding. We identified four initial patches rich in lysines that were not found in ACAP BAR domains. Mutants in which the residues were changed were interrogated for *in vitro* and cellular effects on the actin filaments. From this initial round, we identified lysine-rich cluster at positions 75, 76, and 79 and 159, 163, and 164 that were necessary for efficient actin binding and bundling and, therefore, were possible actin-interaction sites. The contribution of the cluster K159, K163, K164 was less clear. [K159E, K163E, K164E] ASAP1-BAR-PH-His_6_ protein was poorly expressed in bacteria and mammalian cells, and the protein had a lower melting temperature than WT protein, indicating the protein was unstable. The lack of effect of the mutants might be the result of poor stability. Therefore, the importance of this cluster in actin interaction remains ambiguous.

We then probed the area around the initially identified K75, K76, K79 cluster and found that the binding affinity of the [Q72, Y83, N83] cluster was also affected, although to a lesser degree than the primary [K75, K76, K79] site. However, its effect on bundling was as pronounced as the primary site, suggesting that all of these residues participate in cross-linking of the actin filaments. Ongoing structural studies in our laboratory aim to determine spatial arrangement and organization of actin–ASAP1 complexes.

Significance of actin-binding cluster was also assessed in cells. Using transient overexpression of mutants in U2OS cell line, we observed that that the formation of actin microspikes (or actin-rich protrusions) formed by overexpression of the BAR-PH tandem is compromised when residues implicated in actin filament interaction are mutated. Moreover, exogenous expression of the full-length ASAP1 [K75E, K76E, K79E] mutant failed to rescue the effects of ASAP1 knockdown in U2OS cells, further supporting the hypothesis that this cluster is important for the role of ASAP1 in the maintenance of filamentous actin structures. Actin remodeling can have profound effects on behaviors such as signaling, cell cycle, and motility, therefore our future questions are focused on addressing how the effects of ASAP1 on actin control global cellular behaviors.

ASAP subtype Arf GAPs comprise three genes, each with multiple splice variants. ASAP2 and ASAP3 BAR-PH domains are 52 and 58% identical to ASAP1 but are 100% identical or similar in the clusters determined important for actin binding. *In vitro* and in cells, ASAP2 and ASAP3 affected actin filament bundling; however, their cellular functions are not redundant (32, 33) The finding that knockdown of a single ASAP could affect stress fibers suggest that the proteins, although through a similar biochemical mechanism, affect different steps of the process, an area of ongoing work in our laboratory.

When examining the effects of the mutations in ASAP1 on binding to and bundling of F-actin, we found larger differences in the maximum extent of binding than in apparent affinities. The hypothesis we are currently considering is that this a result of examining a highly cooperative process of assembling a lattice comprised of two proteins. There will be a distribution of species including free ASAP1, free F-actin, ASAP1-F-actin, (ASAP1)_2_-F-actin, ASAP1-(F-actin)_2_, (ASAP1)_2_-(F-actin)_2_, etc. A change in affinity of ASAP1 for F-actin might have a large change in distribution while, because of the number of bound species, concentrations for half maximal effects might not the obvious. The change of the distribution in sizes of bundles that we observed is consistent with this hypothesis. We are working on developing approaches to test the hypothesis. Regardless, the mutants we identified affect F-actin bundling *in vitro* and in cells.

ASAP1 is a multi-domain protein with N-BAR, PH, ANK, E/DLPPKP repeats, and SH3 domains that has diverse functions in cells including catalytic (ARF GTPase activating protein), regulation of focal adhesion dynamics (12, 14, 31, 34, 47, 48), promotion of adipogenic and osteogenic differentiation (13, 15), regulation of inflammation (37, 38, 39), and oncogenesis (16, 17, 18). However, the underlying basis connecting these functions remains elusive. The understanding of the biochemical functions of each of the domains, with the accompanying generation of reagents, such as the mutants that are described in this paper, will be necessary to understand the connection of ASAP1's disparate functions.

## Experimental procedures

### Plasmids, antibodies, and reagents

Mutagenic primers were from Eurofins Genomics or Integrated DNA Technologies. All cloning reagents were from New England Biolabs. Plasmids were verified by Sanger sequencing by the CCR Genomics Core (NCI). ASAP1 antibody was previously described (31), ASAP2 and ASAP3 antibodies were from Santa Cruz, His_6_ antibody was from BioLegend, and HA-tag and GAPDH antibodies were from Cell Signaling. Secondary antibodies and fluorescent phalloidins were from Bio-Rad, LI-COR, and ThermoFisher.

Rabbit skeletal muscle globular actin was from Cytoskeleton, Inc. Preformed actin filaments were a kind gift from Dr James R. Sellers (NHLBI).

### Homology modeling

Homology modeling of ASAP1 BAR-PH was performed independently using SWISS-MODEL server with template search and MODELLER using ACAP1 (PDB ID: 4NSW) and APPL1 (PDB ID: 2Q13) as templates and compared to low resolution cryo-EM structure reported in (24). Both models were energy minimized using YASARA server (49), and the energy minimized models were further checked and optimized using MOLProbity (Duke University). Model visualization was performed at every step in PyMOL (Schrödinger, LLC).

Sequence alignment of ASAP BAR-PH regions was performed in ClustalW (50) and visualized using JalView (51).

### Protein expression and purification

Recombinant ASAP1 BAR-PH (aa 1–431) and mutants, ASAP2 BAR-PH (aa 1–397), ASAP3 BAR-PH (aa 1–394), and ACAP1 BARPH (aa 1–370) were expressed in LOBSTR (DE3) RIL (Kerafast) or BL21 (DE3) (Agilent) cells as previously described (20). Protein purification was carried out as described previously (22, 47, 52) with modifications. Plasmids were transformed into BL21(DE3)-RIL or BL21(DE3) LOBSTR and grown in LB supplemented with 0.5% glycerol and 1X metals mix and induced with 0.25 mM IPTG overnight at 18 °C or 3 h at 37 °C. Cell pellets expressing His_6_-tagged constructs were lysed in buffer A (20 mM Hepes pH 7.7, 500 mM NaCl, and 10 mM Imidazole) supplemented with protease inhibitors using three rounds of sonication or using a cell disruptor (Microfluidics). Precleared lysates were passed through Ni-NTA (Qiagen) packed gravity column preequilibrated with Buffer A, and bound His_6_-tagged proteins were eluted with Buffer B (20 mM Hepes pH 7.7, 500 mM NaCl, and 250 mM Imidazole). Alternatively, precleared lysates were fractionated on HisTrap column (GE Healthcare). After elution, the high imidazole buffer was exchanged using Zeba (ThermoFisher) or PD10 (GE Healthcare) buffer exchange columns or purified further using Sephacryl S100 size exclusion gel filtration column on an Akta Pure (GE Healthcare).

All purification steps were done at room temperature.

### Protein thermal stability assay

Purified proteins (2 μM in 50 μl volume) were distributed into thin-walled PCR tubes in three technical replicates and heated in a thermocycler at a temperature gradient of 30 to 63 °C with 3 °C increments, with a 3 min hold at every temperature increment. A room temperature sample was used as a starting reference. The samples were allowed to cool at room temperature for 15 to 20 min and centrifuged to sediment precipitated protein. Remaining soluble fractions (stable protein) were transferred into microcentrifuge tubes, resuspended 1:1 in sample buffer, and separated on 12% SDS-PAGE. Stability experiments were done twice for each mutant. Densitometric quantification of protein bands was performed in ImageJ (NIH), and melting curves were fitted in GraphPad Prism.

### Actin cosedimentation assays

High- and low-speed actin cosedimentation assays were performed according to the manufacturer’s protocol (Cytoskeleton, Inc) as before (20). Prior to incubation with filaments, test protein was spun for 1 h at 150,000*g* at 4 °C to sediment aggregates. For binding experiments, test proteins (2 μM) were incubated with varying concentrations of F-Actin (0–25 μM) in F-actin buffer (5 mM Hepes pH 8.0, 100 mM KCl, 2 mM MgCl_2_, 0.2 mM CaCl_2_, 1 mM ATP, and 1 mM DTT) for 30 min and centrifuged at 150,000*g* for 90 min at 24 °C and/or 4 °C in an ultracentrifuge (Thermo Scientific Sorvall MTX150). For bundling experiments, test proteins were incubated with F-actin as before but spun at 14,000*g* for 30 min at 24 °C in a tabletop centrifuge (Eppendorf 5415R). For analysis of binding, equal volumes from supernatant and pellet fractions were separated on SDS-PAGE, stained with GelCode blue or Coomassie blue, and quantified using ImageJ (NIH).

For fluorescence-based assessment of actin bundling, reactions were prepared as for cosedimentation assays and spotted using large orifice tips on poly-L-lysine coated 12 mm coverslips (Electron Microscopy Sciences). The coverslips were fixed on ice with 4% paraformaldehyde in PBS, stained on ice with rhodamine, Alexa Fluor 488 or 594 phalloidin, and mounted and imaged on a Leica TCS SP8 using LAS X Navigator module. Fifty tiles per coverslip across four coverslips were collected to assess the efficiency of bundling per given experiment.

### Cell culture and transfection

U2OS cell line (ATCC) was maintained in McCoy’s 5A supplemented with 10% heat-inactivated fetal bovine serum and 50 μg/ml penicillin/streptomycin (Gibco). U2OS cells are not on the list of commonly misidentified cell lines. The cell cultures were routinely tested for *mycoplasma* infection using PCR (Sigma-Aldrich).

For knockdown experiments, cells were transfected with 20 nM siRNAs against human ASAP1, ASAP2, ASAP3 (ON-TARGET smart pool), or non-targeting siRNA #4 (Dharmacon) using JetPrime reagent (Polyplus) and analyzed 72 h posttransfection. For overexpression experiments, cells were transfected with pcDNA 3.1(+) plasmids encoding WT or mutant ASAP1 BAR-PH using Lipofectamine LTX and analyzed 24 h later. Stable cell line generation was performed by VectorBuilder. Briefly, synthesized gene inserts of WT and mutant ASAP1a (nm_001362924.1) ORFs (open reading frame) with codons extending the reading frame at the 3′ end with codons encoding a linker of three GGGGS tandems and YPYDVPDYA (the HA epitope) were cloned into a tetracycline-inducible lentiviral backbone using Gateway cloning, verified by sequencing, and transfected into packaging cells for production of lentiviral particles. U2OS cells were transduced with resulting lentivirus for the generation of stable cell lines. For rescue experiments, ASAP1 was knocked down with dicer substrate RNA duplex targeting the 3′UTR (Integrated DNA Technologies) in stable U2OS cell lines using Dharmafect 1 (Horizon Discovery). Expression of empty vector, WT, or mutant ASAP1 was induced by the addition of doxycycline (100 ng/ml) 48 h postdicer transfection.

### Immunofluorescence

Transfected cells were replated in a single cell suspension on fibronectin coated #1.5 German glass coverslips in serum-free Opti-MEM for 5.5 h and fixed in 4% paraformaldehyde in PEM (Pipes/EDTA/MgCl_2_) buffer or PBS (Electron Microscopy Sciences) (33). Free aldehydes were quenched with 100 mM Glycine in PBS, and the cells were permeabilized and blocked with buffer containing bovine serum albumin (BSA) (5% w/v), goat or donkey serum (5% v/v), and saponin (0.2% w/v) in PBS for 1 h, followed by staining with primary antibodies (1 h in blocking buffer) and fluorescently labeled secondary antibodies and/or fluorescently labeled phalloidin (30 min in buffer containing BSA (1% w/v) and saponin (0.1% w/v)). Coverslips were mounted with DAKO mounting medium. Confocal microscopy was performed on Leica TCS SP8 confocal laser scanning microscope using system optimized z-stack parameters with pinhole of 1.00 Airy Unit, an HC PL APO CS 63×/1.4 oil objective, at 700 Hz with bidirectional X, XY = 1024 × 1024 with zoom factor of 1.78, and pixel size of 101.33 × 101.33.

Actin fibers were quantified with the Ridge Detection plugin in ImageJ (30). The number of fibers of length greater than 2.2 μm was determined as a cut-off to distinguish between cells with and without stress fibers based on histograms of fiber length comparing cells treated with control siRNA or siRNA targeting ASAP1, which had previously been established as cells with and without stress fibers (20, 22, 24). Approximation of stress fiber alignment was performed in ImageJ using FibrilTool plugin (35).

### Immunoblotting

Samples were separated by SDS-PAGE (Bio-Rad) and transferred onto nitrocellulose membranes. After blocking (5% w/v BSA in 0.1% (v/v) Tween-20 in TBS), the membranes were incubated overnight at 4 °C with primary antibodies diluted in blocking buffer. Membranes were washed thrice in TBS +0.1% Tween-20 (v/v) and incubated with horseradish peroxidase-conjugated (Bio-Rad) or IRDye-labeled (LI-COR) secondary antibodies for 1 h. Membranes then were washed thrice in TBS +0.1% Tween-20 (v/v), once in plain TBS and processed with SuperSignal West Dura chemiluminescent substrate (ThermoFisher) using ChemiDoc Imaging System (Bio-Rad) or LI-COR imaging system. Immunoblot images were quantified using ImageLab v5.2.1 (Bio-Rad) and ImageStudioLite (LI-COR).

### Statistical analysis

Statistical analyses were carried out using Prism 8 (GraphPad). Comparison of two groups was carried out using Student’s *t* test, and comparison of data sets with more than two groups was carried out using ANOVA. Alpha was set to 0.05 for all experiments. The values represented are mean ± s.e.m., unless otherwise noted in the figure legends. For figures describing cell biology experiments, “N” indicates number of cells per treatment group per experiment, unless otherwise noted.

## Data availability

All data are contained within the article.

## Supporting information

This article contains supporting information.

## Conflict of interest

The authors declare that they have no conflicts of interest with the contents of this article.

## Footnotes

† Unpublished observations.

‡ The cDNA for ASAP1a (nm_001362924.1), which has a 54-amino acid exon in the proline rich region not present in ASAP1b, was expressed under control of the tet promoter. Endogenously expressed protein was primarily ASAP1b. The additional 54 amino acid residues with the tag account for the difference in migration of the ectopically expressed and endogenous ASAP1.
