# Supplementary material for: A lysine-rich cluster in the N-BAR domain of ARF GTPase-activating protein ASAP1 is necessary for binding and bundling actin filaments
Source: J Biol Chem. 2022 Feb 8;298(3):101700. doi: 10.1016/j.jbc.2022.101700 (PMC8902617; doi:10.1016/j.jbc.2022.101700)

Supporting Information

Figure Legends

Figure S1, related to Figure 2. A. Thermal stability assay of ASAP1 BAR-PH mutants. Alanine scanning or charge reversal mutants of ASAP1 BAR-PH were heated at the indicated temperature and remaining soluble (stable) fractions were resolved on SDS-PAGE and quantified. Gels representative of at least two experiments done in triplicates are shown for each mutant. Resulting melting curves were fitted using Prism and are shown as two separate graphs for clarity of presentation. **B.** Summary of calculated T_m (melting temperature) for each protein as a mean ± SEM of all experiments. RT- room temperature.

Figure S2, related to Figure 5. Complementation with ASAP1 [K75E, K76E, K79E] does not rescue the effects of ASAP1 downregulation on actin stress fibers. U2OS cells were stably transduced with tet-inducible empty vector, full-length wild type (HA-ASAP1) or HA-ASAP1 [K75E, K76E, K79E] lentiviruses and transfected with control (diCtrl) DICER substrate RNA duplex or diRNA against the 3'UTR region of human ASAP1 (diASAP1). After 24 hours, expression of the empty vector or ASAP1 was induced or not with doxycycline (100 ng/mL) for 48 hours. Cells were plated on fibronectin in serum-free media, fixed and stained with fluorescent phalloidin for F-actin and imaged with a confocal microscope. Z-projection of the images were analyzed with the Ridge Detector plugin in Fiji to quantify the number of actin filaments greater than 2.2 μm in length in each cell. The results are presented in the graphs, with each point representing the number of filaments in a cell under the indicated condition. Each graph has data from an individual experiment. The summarized data are presented in Figure 5.

Figure S3, related to Figure 6. Results of ClustalW alignment of BAR-PH regions of human and mouse ASAP subtypes was visualized in JalView. Amino acids examined in this study are in boxes.

Figure S1. Gasilina et al.

A

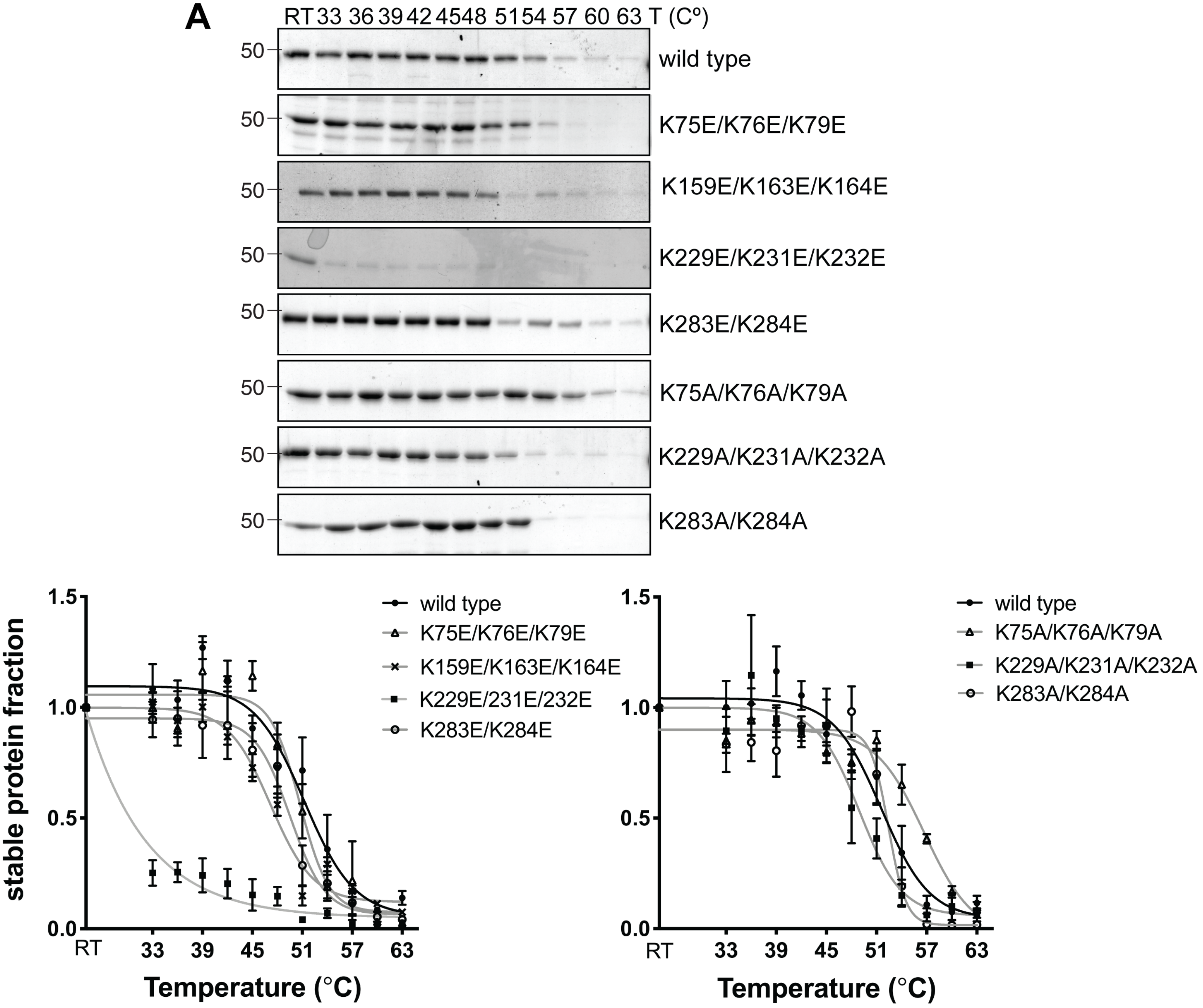

B

protein	Tm
wild type	51.2 ± 0.9
K75E/K76E/K79E	50.7 ± 0.6
K159E/K163E/K164E	47.2 ± 0.5
K229E/K231E/K232E	not determined
K283E/K284E	49.5 ± 0.8
K75A/K76A/K79A	56.7 ± 4.5
K159A/K163A/K164A	no expression
K229A/K231A/K232A	49.0 ± 1.0
K283A/K284A	52.4 ± 0.5
K101/K105A	no expression

Figure S2. Gasilina et al.

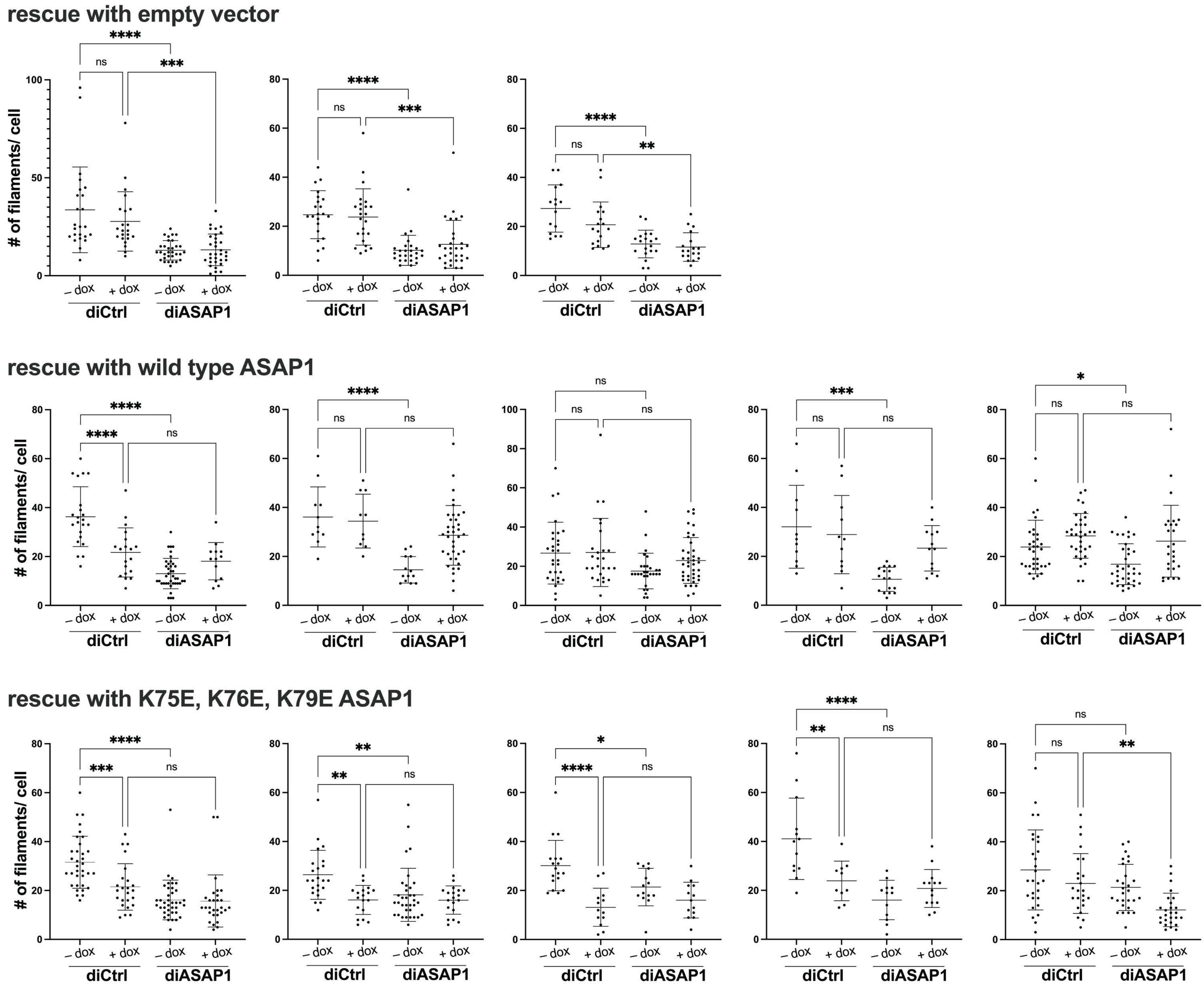

Figure S3 Gasilina et al.

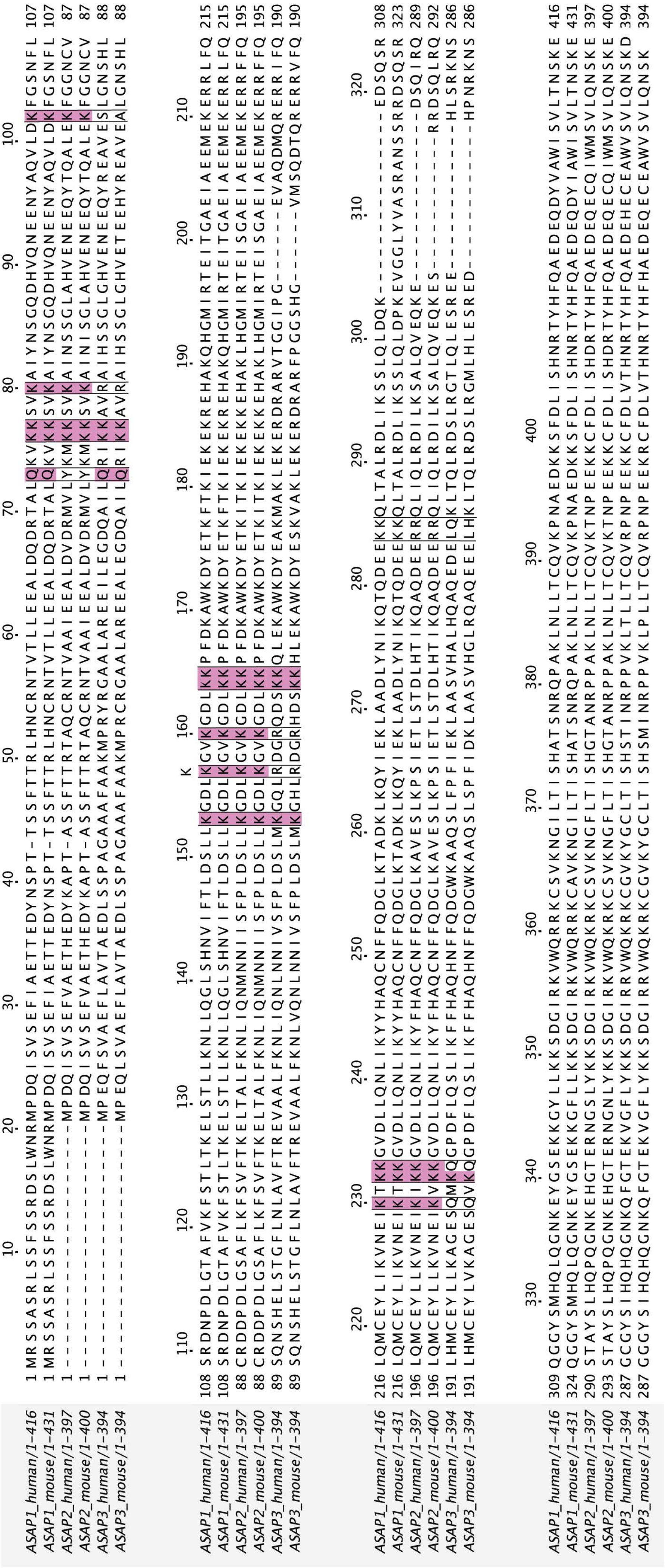

Supplement: Supplemental Figures S1–S3 [file mmc1.pdf]
